# Comparative Tandem Mass Tag-Based Quantitative Proteomics Analysis of Liver Against Chronic Hypoxia: Molecular Insights Into Metabolism in Rats

**DOI:** 10.1089/ham.2022.0003

**Published:** 2023-03-17

**Authors:** Jin Xu, Shenhan Gao, Mingyuan Xin, Wenjie Chen, Kaikun Wang, Wenjing Liu, Xinzong Yan, Sinan Peng, Yanming Ren

**Affiliations:** ^1^ Department of Medicine, Qinghai University, Xining, China.; ^2^ Department of Ecology, Qinghai University Plateau Biology Jing Ying Class, Grade 19, Xining, China.; ^3^ Department of Medicine, Qinghai University Clinical Medicine Class 1, Grade 19, Xining, China.; ^4^ Department of Medicine, Qinghai University Clinical Medicine Class 6, Grade 20, Xining, China.; ^5^ Department of Medicine, Qinghai University Clinical Medicine Class 3, Grade 20, Xining, China.; ^6^ Department of Medicine, Qinghai University the Graduate Student of Foundation Medical of 2020, Xining, China.; ^7^ Department of Medicine, Qinghai University the Graduate Student of Foundation Medical of 2021, Xining, China.; ^8^ Department of Mechanical Engineering, Qinghai University Material Forming and Control Engineering Class, Grade 20, Xining, China.

**Keywords:** chronic hypoxia, liver, proteomics, TMT

## Abstract

Xu, Jin, Shenhan Gao, Mingyuan Xin, Wenjie Chen, Kaikun Wang, Wenjing Liu, Xinzong Yan, Sinan Peng, and Yanming Ren. Comparative tandem mass tag-based quantitative proteomics analysis of liver against chronic hypoxia: molecular insights into metabolism in rats. *High Alt Med Biol*. 24:49–58, 2023.

**Objective:**

Using a metabolomic approach, we uncovered key regulators in metabolism from tandem mass tag (TMT)-based proteomic analysis in animals chronically exposed to hypoxia.

**Methods:**

Sixteen Sprague–Dawley rats (*n* = 8 per group) were exposed to chronic normoxia or hypoxia (380 mmHg corresponding to a simulated altitude of 5,500 m) for 35 consecutive days. Hypoxia-induced alterations in metabolic pathways were analyzed from TMT-based proteomic analysis, complemented by western blot validation of key regulators.

**Results:**

We profiled biochemical parameters and serum lipids, found that serum alanine aminotransferase and blood glucose were not significantly changed due to chronic hypoxia. However, serum triglycerides, total cholesterol, high-density lipoprotein, and low-density lipoprotein (LDL) were significantly affected by chronic hypoxia. And the levels of LDL nearly doubled (*p* < 0.05) after hypoxia exposure for 35 days. Through Kyoto Encyclopedia of Genes and Genomes classification, we found several metabolic pathways were enriched, including lipid metabolism, cofactor and vitamin metabolism, amino acids metabolism, carbohydrate metabolism, and energy metabolism. To explore the potential functions of proteins in metabolic pathways that become a coordinated shift under chronic hypoxic conditions, Gene Ontology and pathway analysis were carried out on differentially expressed proteins. As the co-expression network shown in Figure, we identified the most significant differentially expressed proteins after chronic hypoxic changes in the livers of rats. Furthermore, we validated the gene expression profiles at the protein level using western blot. Results of western blot were in accordance with our quantitative polymerase chain reaction findings. The levels of fatty acid synthase and aquaporin 1 were significantly downregulated after 35 days and the levels of ATP citrate lyase, 2′-5′-oligoadenylate synthetase 1A, aldehyde dehydrogenase 2, and Ras-related protein Rap-1A were significantly upregulated after 35 days.

**Conclusions:**

Although this study cannot completely account for all the molecular mechanisms in rats, we provide a good analysis of protein expression and profiling of rats under chronic hypoxia conditions.

## Introduction

Generally, hypoxia is triggered by a shortage of oxygen supply, which leads to a global transcriptional and translational change in most tissues in humans (Zhou et al, 2018). Hypoxia is coordinated shift, when oxygen delivery is disrupted or reduced, the organisms will develop numerous adaptive mechanisms to facilitate cells survived in the hypoxic condition (Chen et al, 2020). The liver is involved in most metabolic processes and acts as a key player in the maintenance of metabolic homeostasis (Conotte et al, 2018; Scha Dd E et al, 2017). Although there are many reports exploring hypoxia-related factors, similar to hypoxia-inducible factors in mammals (Chiu et al, 2019), the intracellular mechanisms involved in liver against chronic hypoxia have not been well elucidated or reported.

Chronic hypoxia-related diseases usually occur in high-altitude areas. In China, chronic hypoxia sometimes even results in states of serious illness impacting different organs, such as high-altitude pulmonary edema and high-altitude cerebral edema. Hypoxia can also result in chronic intermittent hypoxia-mediated renal sympathetic nerve activation in hypertension and cardiovascular disease (Bhagwani et al, 2020). Even worse, chronic hypoxia in premature infants can result in serious lung injury (Kang et al, 2018). Thus, the diseases triggered by chronic hypoxia could result in illness in a large probability, which might bring more impairment to the patients.

It is well known that the liver is a central organ that metabolizes glycogen, lipids, and supplies energy-producing substrates to peripheral tissues to maintain their function in different conditions, including hypoxia and chronic hypoxia. For example, variations in lipid metabolism caused by hypobaric chronic hypoxia has been shown to involve activation of a number of key enzymes and metabolic pathways (Varun et al, 2018). A genome-wide scan in adipocytes revealed that peroxisome proliferator activated receptor alpha and angiopoietin-like protein 4 (ANGPTL4) were significantly associated with expression of several genes encoding proteins that control fatty acid metabolism (González-Muniesa et al, 2011; Yin et al, 2009).

Quantitative proteomics is a cutting-edge technique for quantifying the amount of protein present in a sample. Mass spectrometry (MS)/MS-based proteomics has become a popular technique, owing to the ability to accurately quantify >9,000 proteins across multiple samples within a single experiment (Moulder et al, 2018). Proteomics analyses are powerful tools in areas such as the investigation of novel biomarkers, unveiling biological processes, as well as identifying aberrant expression of proteins. These tools are available for researchers to use for tracking changes across thousands of proteins with simple processes. As such, it is possible for us to investigate molecular changes during chronic hypoxia challenges in rats by utilizing these techniques.

To investigate the mechanisms at work within the liver under chronic hypoxia conditions, we used animal experiments and tandem mass tag (TMT)-based proteomics to identify aberrant expression of proteins in the liver of chronic hypoxia-treated rats. Finally, by taking all the results together, we generated a speculative regulation network of chronic hypoxia-induced metabolic changes in rats.

## Methods

### Chronic hypoxia rat models

In this study, Sprague–Dawley male rats were randomly allocated into two groups (eight animals per group) containing normal rats (Control) and rats exposed to chronic hypoxia for 35 days (Hypoxia). Rats in the chronic hypoxia group were exposed to a simulated altitude atmosphere with 5,500 m (380 mmHg), implemented by a FLYDWC50-1C low-pressure hypoxic experimental cabin (Guizhou Fenglei Air Ordnance Ltd., Guizhou, China).

During breeding and experimental procedures, animals in both groups were housed in the same density per cage at a controlled ambient temperature of 25 ± 2°C and 50 ± 10% relative humidity with a 12-hour light/12-hour dark cycle. Rats were given standard rodent chow and water *ad libitum*. After overnight fasting, rats were sacrificed under anesthesia with 10% chloral hydrate (0.4 mL/100 g body weight, intraperitoneal). The right lobe of the liver was snap-frozen in liquid nitrogen and then stored at −80°C until analysis. The research protocol was approved by the Human Subject Protection Committee at the Qinghai University School of Medicine (Xining, China) (IACUC Issue No. 2019-ZJ-876).

### RNA preparation and quality control

Total RNA was extracted by Trizol using the manufacture's protocol (Invitrogen Co. Ltd.). Quality control of extracted RNA was subsequently conducted using a Thermo Nanodrop 3000. Microarray analysis was only performed on quality RNA with a standard: 1.8 < A260/A280 < 2.2 by Thermo Nanodrop 3000.

### Plasma and hepatic lipid profiles

Plasma lipids were measured on days 0 and 35. Plasma low-density lipoprotein (LDL), triglycerides (TG), total cholesterol (CHO), high-density lipoprotein (HDL), and very LDL were measured using the Vitros DT60 II Chemistry System (Johnson & Johnson, Minneapolis, MN). The liver samples were homogenized using a Stir-Pak^®^ (Barrington, IL). Total CHO and TG were extracted in a chloroform-methanol mixture (2:1) and measured with the same Vitros DT60 II Chemistry System.

### Protein extraction

Proteins were extracted using a protein extraction kit (Promega, USA), followed by quantification with a bicinchoninic acid (BCA) Protein Assay Kit (Bio-Rad, USA); at least 160 mg of protein was collected per liver sample. Samples were then run on a sodium dodecyl sulfate-polyacrylamide gel electrophoresis (SDS-PAGE) gel electrophoresis and Coomassie bright blue staining was used to indicate the location of protein on the gel. Finally, the protein suspension was digested with trypsin (Promega) in NH_4_HCO_3_ at 37°C overnight, and the yielded proteins were filtered. Nearly 160 mg of protein was collected.

### TMT labeling of proteins and high pH reverse phase fractionation

TMT reagents were used for the labeling of proteins according to the manufacturer's instructions (Thermo Fisher Scientific), samples were collected and kept on ice for liquid chromatography (LC)-MS analysis.

### LC-MS analysis

For LC-MS/MS analysis, each sample was injected once for a total of 16 times. The high-performance liquid chromatography liquid phase system was used for phase separation. Proteins were analyzed by the Q Exactive-Plus (QE-Plus) software (Thermo Scientific, Waltham, MA). The resolution of the first-level MS was 65,000 at m/z 200, the first-level Maximum injection time was set to 49 ms, automatic gain control was set to 1E6, and the resolution of the second-level MS was set to 35.

### Bioinformatics analysis

R packages and the statistical computing software were used to analyze the bioinformatics data. Proteins were screened with the cutoff ratio fold-change of >1.30 and *p*-values <0.05. Hierarchical clustering was used to visualize protein level. Gene Kyoto Encyclopedia of Genes and Genomes (KEGG), Gene Ontology (GO) enrichment, and protein interaction network analysis were done with R packages (Cran Inc., USA).

### Western blotting analysis

Fresh liver tissue from each animal was homogenized in homogenization buffer, followed by centrifugation at 12,000 *g* for 15 min. The supernatant was removed and protein quantification was performed using a BCA assay. Equal amounts of protein (60 μg) were resolved on SDS-PAGE gels and transferred to a polyvinylidene fluoride membrane. The membrane was blocked using 5% nonfat dry milk in Tris buffered saline with Tween-20 (TBS-T) buffer for 2 hours. The membrane was incubated with primary antibody (Abcam, USA) overnight and washed three times for 5 minutes each with TBS-T, followed by incubation with the secondary antibody (1:3,000; Santa, Inc.) for 1 hour and three additional washes for 5 minutes each with TBS-T. The blots were imaged using a Tiangen chemiluminescence system (Tiangen, China) and glyceraldehyde-3-phosphate dehydrogenase was selected as the internal reference.

### Data analysis and statistics

The results were entered into a database and were analyzed using Statistical Product and Service Solutions (SPSS) 21.0 (SPSS, Inc., Chicago, IL). The mean, standard deviation (SD), standard error, and confidence interval were calculated for each parameter. Data were presented as mean ± SD. Student's *t*-test was applied, when appropriate, to determine the statistical significance of the differences. Pearson's correlations were also performed. The results were considered significant when the *p*-value was <0.05.

## Results

### General workflow and summary of this study and plasma lipid metabolism profiles

**FIG. 1.**
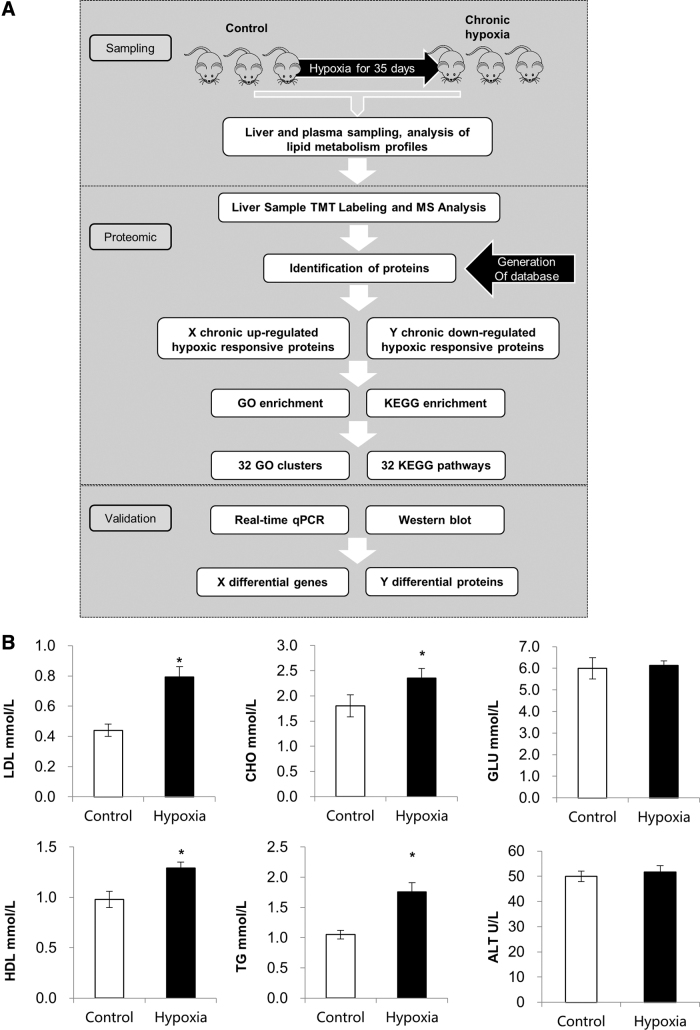
General workflow and summary of this study and plasma lipid metabolism profiles. (**A)** General workflow details of each process are shown. **(B)** Plasma lipid metabolism profiles including LDL, CHO, HDL, GLU, TG, and ALT levels are shown. Student's *t*-test, paired tail, *p < 0.05. ALT, alanine aminotransferase; CHO, cholesterol; GLU, glucose; GO, Gene Ontology; HDL, high-density lipoprotein; KEGG, Kyoto Encyclopedia of Genes and Genomes; LDL, low-density lipoprotein; MS, mass spectrometry; qPCR, quantitative polymerase chain reaction; TG, triglycerides; TMT, tandem mass tag.

To explore the influence of chronic hypoxia on lipid metabolism, we first created a chronic hypoxia animal model using Sprague–Dawley male rats according to a previous report. In brief, rats were randomly allocated to two groups (eight animals per group) containing normal rats and rats exposed to chronic hypoxia for 35 days. Our overall workflow is shown in Figure 1A. Next, we profiled biochemical parameters and serum lipids on day 35. Serum alanine aminotransferase (ALT) and blood glucose (GLU) were not significantly changed due to chronic hypoxia. However, serum TG, total CHO, HDL, and LDL were significantly affected by chronic hypoxia (Fig. 1B). Interestingly, the levels of LDL nearly doubled (*p* < 0.05) after hypoxia exposure for 35 days.

Above all, our lipid profile analysis has demonstrated that chronic hypoxia may have a significant impact on lipid metabolism.

### KEGG pathway enrichment analyses of all differentially expressed proteins (*p*-value of Fisher's exact test <0.05)

**FIG. 2.**
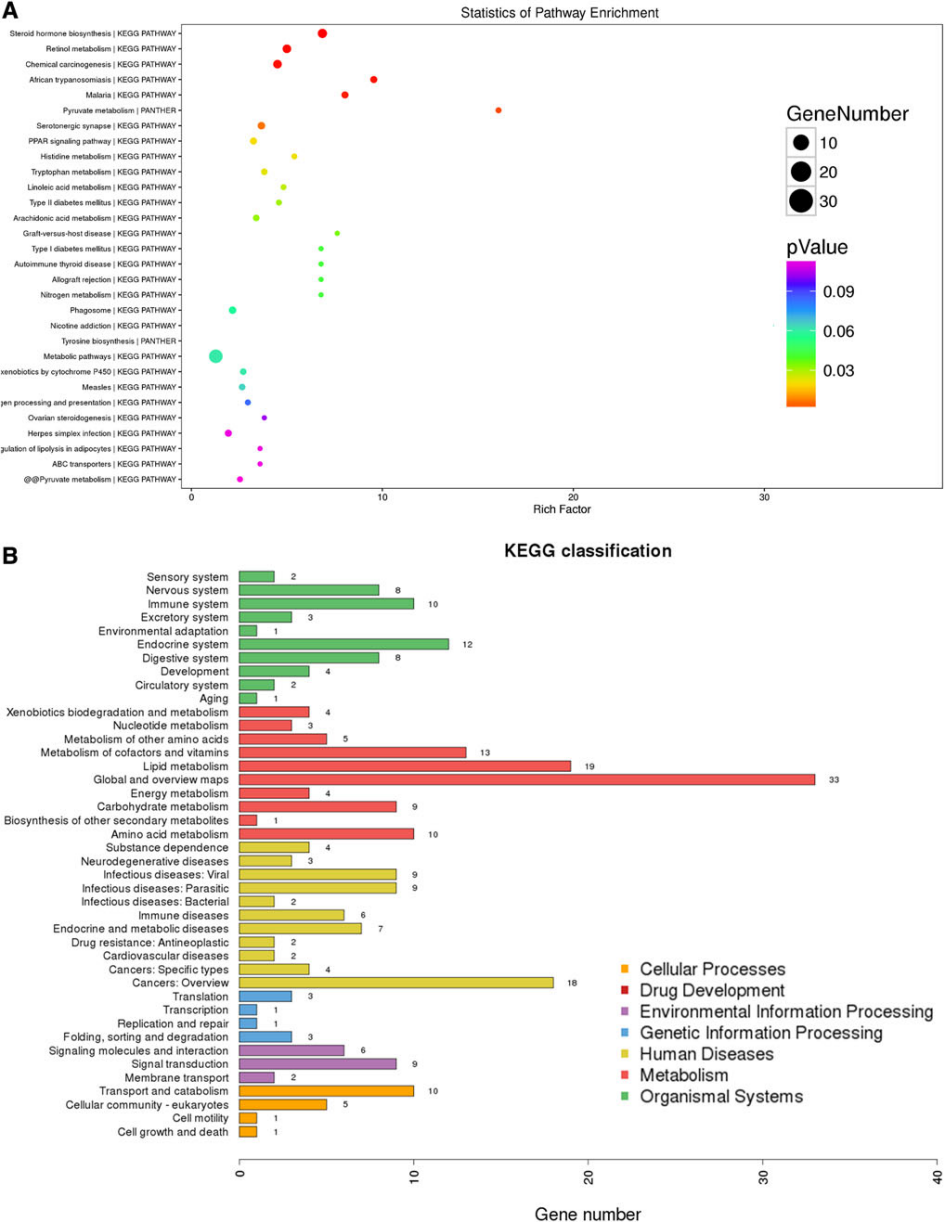
KEGG pathway enrichment analyses. **(A)** KEGG analysis of all significantly regulated proteins (*p*-value of Fisher's exact test <0.05). **(B)** KEGG classification of all significantly regulated proteins based on total gene numbers (*p*-value of Fisher's exact test <0.05).

Protein was profiled from the experimental groups. KEGG was used to identify canonical pathways. In our survey of existing data, the protein precursors mainly involved the following pathways: metabolic pathways, steroid hormone biosynthesis, retinol metabolism, chemical carcinogenesis, PPAR signaling pathway, and glutathione metabolism (Fig. 2A). Furthermore, through KEGG classification, we found several metabolic pathways were enriched, including lipid metabolism, cofactor and vitamin metabolism, amino acids metabolism, carbohydrate metabolism, and energy metabolism (Fig. 2B). The aforementioned findings support that chronic hypoxia significantly affects metabolic pathways in the livers of rats.

### GO enrichment analysis of all differentially expressed proteins (*p*-value of Fisher's exact test <0.05)

**FIG. 3.**
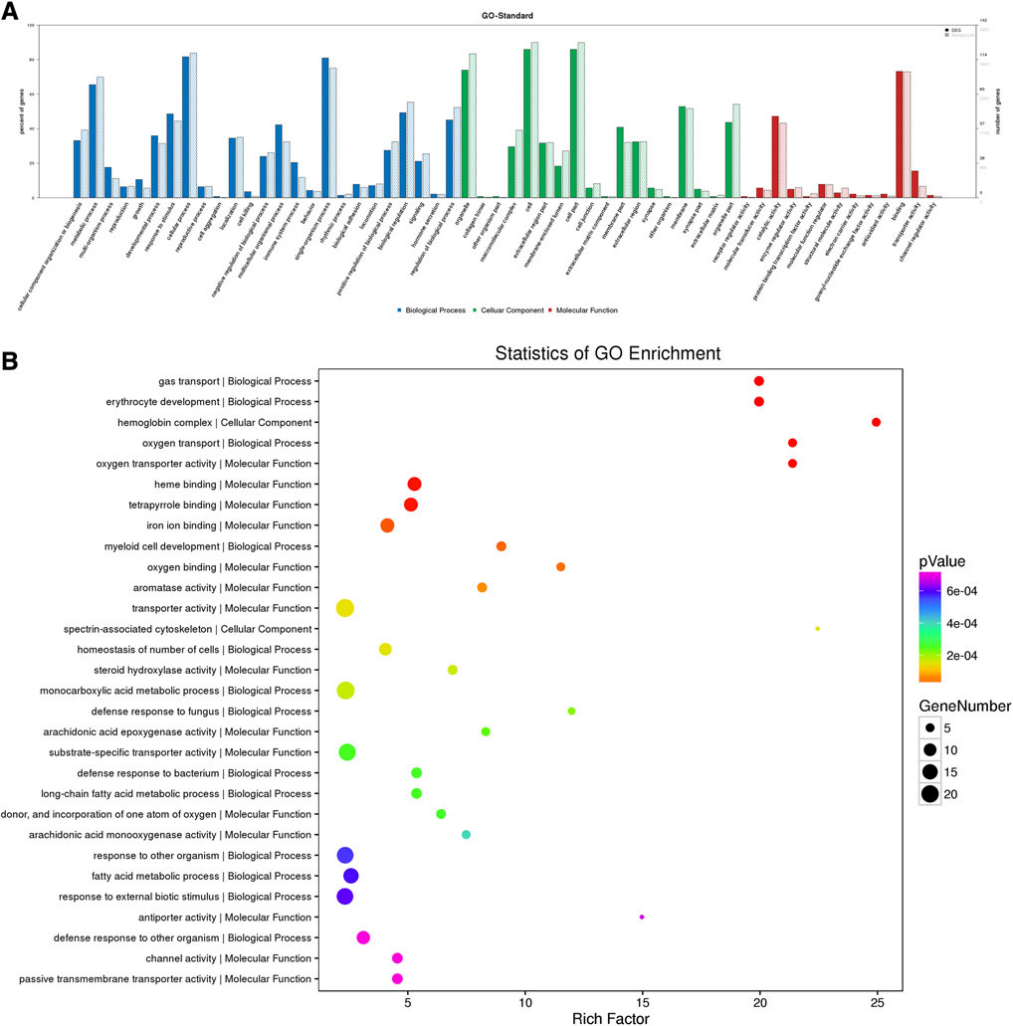
GO enrichment analysis. **(A)** GO analysis of all differentially expressed proteins (*p*-value of Fisher's exact test <0.05). **(B)** Statistics of GO enrichment based on rich factor (*p*-value of Fisher's exact test <0.05).

To explore the potential functions of proteins in metabolic pathways that become a coordinated shift under chronic hypoxic conditions, GO and pathway analysis were carried out on differentially expressed proteins. In a search of the cellular component category, we found that the extracellular region part, membrane-enclosed lumen, macromolecular complex, extracellular region, membrane, cell part, and cell junction were the most populated subcategories (Fig. 3A). For molecular function, we found that catalytic activity, binding, transporter activity, molecular function regulation, structural molecule activity, and transporter activity were most significantly enriched (Fig. 3A).

For biological processes, we found that cellular process, single-organism process, biological regulation, metabolic process, positive regulation of biological process, response to stimulus, and developmental process were the most abundant subcategories (Fig. 3A). Furthermore, through GO enrichment classification, we found several processes were mainly classified as being metabolic, including carboxylic acid metabolic process, response to other organism, response to external biotic stimulus, response to biotic stimulus, organic acid biosynthesis process, oxidoreductase activity process, and oxygen-containing compound process (Fig. 3B). The aforementioned findings support that chronic hypoxia significantly affects metabolic processes and resulted in activation of various responses in the livers of rats.

### Co-expression network of differentially expressed proteins

**FIG. 4.**
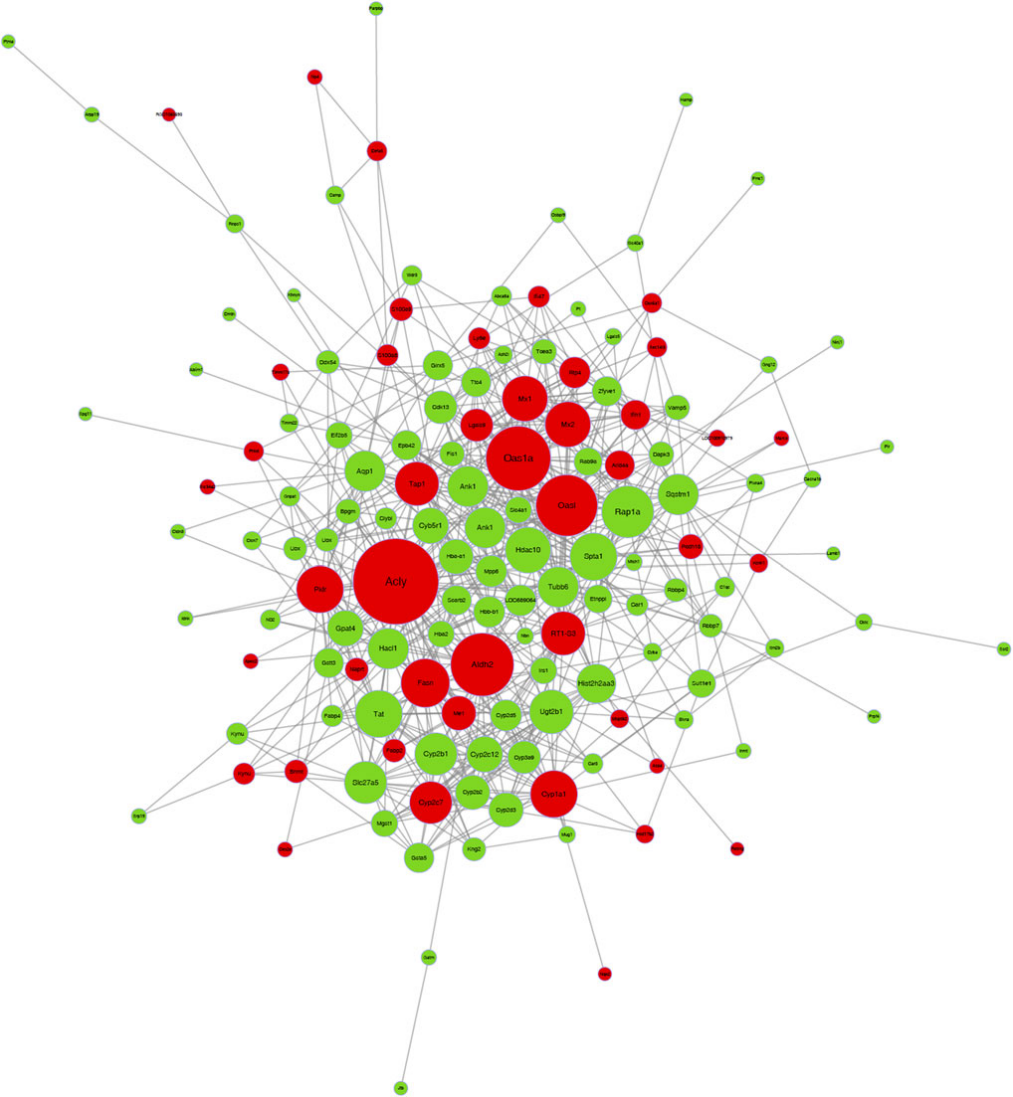
Co-expression network of differential expressed proteins are clearly shown; *red bubble* and *green bubble* indicated the upregulated and downregulated proteins in acute hypoxia challenged group, respectively (*p*-value of Fisher's exact test <0.05).

As the co-expression network shown in Figure 4, we identified the most significant differentially expressed genes after chronic hypoxic changes in the livers of rats. Among them, fatty acid synthase (FASN), ANGPTL4, recombinant sequestosome 1 (SQSTM1), solute carrier family 24 member 1 (SLC24A1), aldehyde dehydrogenase 2 (ALDH2), ATP citrate lyase (ACLY), cytochrome P450 1A1 (CYP1A1), cytochrome P450 1A2, cytochrome P450 4A8, Ras-related protein Rap-1A (RAP1A), palmitoylated membrane protein 6, acyl-CoA synthetase medium-chain family member 2, recombinant human pyruvate kinase L/R, 2′-5′-oligoadenylate synthetase 1 (OAS1), fatty acid binding protein 2, protocadherin 18, S100 calcium binding protein A8, and solute carrier family 4 member A1 formed the core of co-expression network, which were in close relationship with fatty acid metabolism (Table 1).

**Table 1.** Co-Expression Network of Differentially Expressed Proteins

The core of co-expression network
Fatty acid synthase (FASN)
Angiopoietin-like 4 (ANGPTL4)
Sequestosome 1 (SQSTM1)
Solute carrier family 24 member 1 (SLC24A1)
Aldehyde dehydrogenase 2 (ALDH2)
ATP citrate lyase (ACLY)
Cytochrome P450 1A1 (CYP1A1)
Cytochrome P450 2A2 (CYP1A2)
Cytochrome P450 4A8 (CYP4A8)
Ras-related protein1 (RAP1A)
Membrane penetrating particle 6 (MPP6)
Acyl-CoA synthetase medium-chain family member2 (ACSM2)
Recombinant human pyruvate kinase (PKLR)
2′-5′-Oligoadenylate synthetase-like (OASL)
Fatty acid binding protein 2 (FABP2)
Protocadherin 18 (PCDH18)
S100 calcium binding protein A8 (S100A8)
Solute carrier family 4 member A1 (SLC4A1)

### Validation of differentially expressed proteins by real-time quantitative polymerase chain reaction

**FIG. 5.**
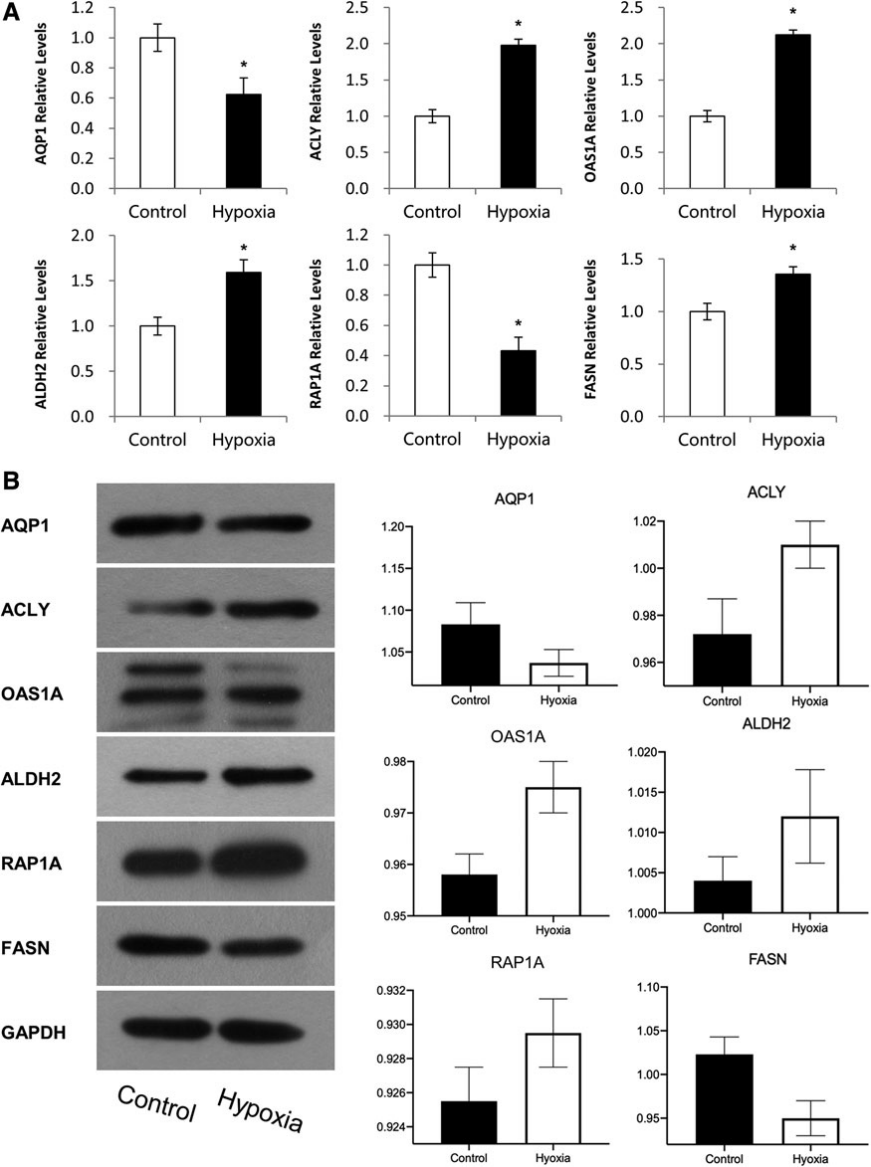
Validation differentially expressed proteins for control and hypoxia samples by real-time qPCR. **(A)** Real-time qPCR validation of FASN, ANGPTL4, SLC24A1, ALDH2, CYP1A1, and SQSTM1. GAPDH was used as internal control. Each experiment was conducted in triplicate. Student's *t*-test, paired tail, *p < 0.05. **(B)** Western blot validation of FASN, ANGPTL4, SLC24A1, ALDH2, CYP1A1, and SQSTM1. GAPDH was used as internal control. Each experiment was conducted in triplicate; the shown figure was a typical replicate of the three experiments.

After analyzing the differentially expressed genes with GO, KEGG, and co-expression network, we focused on the fatty acid metabolism, which was the most enriched pathway in response to chronic hypoxia stimulus. As shown in Figure 5A, we validated the gene expression of FASN, ANGPTL4, SLC24A1, ALDH2, CYP1A1, and SQSTM1 with real-time quantitative polymerase chain reaction (qPCR). In Figure 5B, we further validated the gene expression profiles at the protein level using western blot. Results of western blot were in accordance with our qPCR findings. The levels of FASN and aquaporin 1 (AQP1) were significantly downregulated after 35 days and the levels of ACLY, OAS1A, ALDH2, and RAP1A were significantly upregulated after 35 days.

### A schematic model of sustained reactions to chronic hypoxia conditions in the livers of rats

**FIG. 6.**
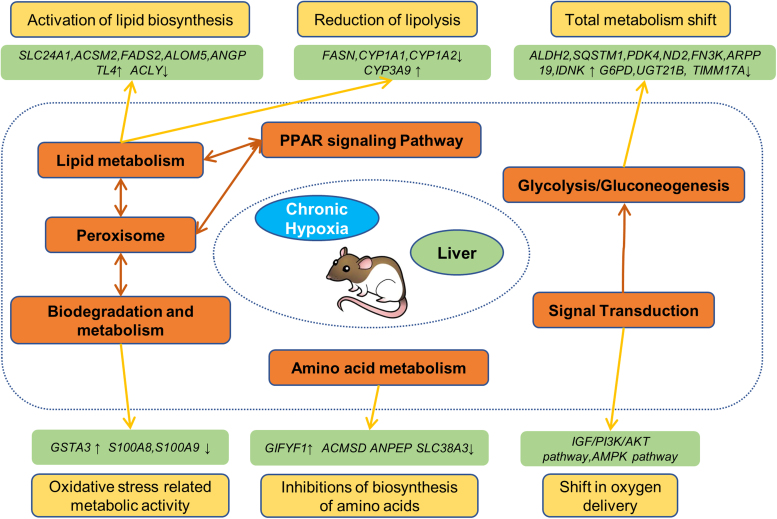
A schematic model of acute reactions in the liver of rats by acute hypoxia. In this figure, we concluded the results and generated the schematic model. For abbreviations and explanations, please see the text.

Based on the bioinformatic analysis and validation data, we constructed a schematic model of the responses that occur in rat livers during hypoxia stimulation. In the graphic view (Fig. 6), we show the network between hypoxia stimulation, metabolic pathways, and the regulatory effects.

## Discussion

We used animal experiments and TMT-based proteomics analyses to identify the changes to metabolism in response to chronic hypoxia. We found that multiple chronic hypoxia responsive proteins in various biological pathways were activated. Serum ALT and aspartate transaminase have been previously described as markers for hepatocyte injury. In our study, we found that ALT was not significantly changed due to chronic hypoxia. These data were consistent with the results of Kamal et al (2017), which were that a sedentary group compared with an exercise group had no difference in ALT. Under hypoxia (10% oxygen for 4 weeks), the contents of TG, HDL, and LDL have been previously shown to increase (Sugimoto et al, 2021). Our research also found that serum TG, CHO, HDL, and LDL were higher in chronic hypoxia than in control groups. These findings support the notion that hypoxia does not result in hepatocyte injury; however, it does influence liver lipid metabolism.

Another finding was the identification of protein precursors involved the following pathways: metabolic pathways, steroid hormone biosynthesis, retinol metabolism, chemical carcinogenesis, PPAR signaling pathway, glutathione metabolism, and so on. (Fig. 2A). Furthermore, through KEGG classification, we found several metabolic pathways, including lipid metabolism, cofactor and vitamin metabolism, amino acids metabolism, carbohydrate metabolism, and energy metabolism (Fig. 2B). The aforementioned findings reveal that chronic hypoxia has significant effects on metabolic pathways in the livers of rats. In a study by Sun et al (2021) (1% oxygen for 24 hours), their results found that hypoxia resulted in differential regulation of 373 messenger RNAs (mRNAs), 334 long noncoding RNAs, 71 circular RNAs, and 33 microRNAs, which contained some of the same genes and pathways identified in our research.

We carried out GO and pathway analysis based on of the differential expression of proteins. We found that the seven most populated subcategories in the search of the cellular component category were extracellular region part, membrane-enclosed lumen, macromolecular complex, extracellular region, membrane, cell part, and cell junction, that the six most significantly enriched subcategories for molecular function were catalytic activity, binding, transporter activity, molecular function regulation, structural molecule activity, and transporter activity, and that the eight most abundant subcategories for biological process were cellular process, single-organism process, biological regulation, metabolic process, positive regulation of biological process, response to stimulus, and developmental process.

Furthermore, we found several processes are mainly classified as metabolic. The study of Li et al (2021) (10% oxygen for 48 hours): the unigenes of rats were individually assigned to the following GO categories, respectively: biological process, cellular component, and molecular function. In our study, ACLY, OAS1A, ALDH2, and FASN were upregulated, and AQP1 was downregulated. In the research of Lee et al (2017) (5% CO_2_ for 24 hours), hypoxia induced upregulation of FASN mRNA expression. Rutkovskiy et al (2013) found that hypoxia (0.5% oxygen for 4 hours) in mice reduced AQP1 mRNA expression (*p* < 0.0001).

Hypoxia, mainly through early mitochondrial function preservation, prevents energetic failure and reactive oxygen species production, improving brain glucose utilization (Sanches et al, 2021). Based on bioinformatic analysis and validation data, we were able to construct a schematic model of the reactions in the livers of rats in response to hypoxia stimulation. In the graphic view, we show that the network overlap between hypoxia stimulation, metabolism pathways, and the regulation effects has important implications for metabolic pathways and regulation under hypoxic conditions.

## Conclusion

Although this study cannot completely account for all the molecular mechanisms in rats, we provide information on protein expression and profiling of rats under chronic hypoxia conditions. The observed hypoxia-related changes in the liver proteome of the rats can help to understand hypoxia-related responses.
